# Assessing the Safety of Combined Therapy, *Croton membranaceus* and Tamsulosin, in the Self-Prescribed Treatment Protocol for Benign Prostatic Hyperplasia

**DOI:** 10.1155/jt/5574491

**Published:** 2025-02-28

**Authors:** Cosmos Gborsong, George A. Asare, Robert A. Ngala, Christian Obirikorang, Josephine Ablakwa, Bernice Asiedu, Samuel Adjei, Osei Afriyie, Daniel Afriyie, Mokbul Hossain, Munmun Pervin, Md. Mahmudul Alam, Mst. Antora Akter, Mohammed Habibur Rahman

**Affiliations:** ^1^Department of Molecular Medicine, School of Medical Sciences, Kwame Nkrumah University of Science and Technology, Kumasi, Ghana; ^2^Department of Medical Laboratory Sciences, School of Biomedical and Allied Health Sciences, University of Ghana, Accra, Ghana; ^3^Department of Animal Experimentation, Noguchi Memorial Institute of Medical Research, University of Ghana, Accra, Ghana; ^4^Department of Pathology, Ghana Police Service, Accra, Ghana; ^5^Department of Pathology, Faculty of Veterinary Science, Bangladesh Agricultural University, Mymensingh, Bangladesh; ^6^Department of Surgery and Obstetric, Faculty of Veterinary Science, Bangladesh Agricultural University, Mymensingh, Bangladesh

**Keywords:** benign prostatic hyperplasia, combination therapy, *Croton membranaceus*, tamsulosin

## Abstract

**Background:** The safety of combining tamsulosin (an allopathic drug) and *Croton membranaceus* aqueous extract, a medicinal plant for managing benign prostatic hyperplasia (BPH), was investigated.

**Methods:** The roots of *CM* were used and processed into a water extract by maceration and decoction. Thirty-five Sprague Dawley rats were divided into seven groups of five rats each. Groups 2–7 were orchidectomy/testosterone injections BPH-induced. Group 1 was designated as the control group. Group 2 was designated as the model group (untreated). Group 3 was treated with 0.03 mg/kg b.wt. of tamsulosin. Group 4 received 30 mg/kg b.wt. of *CM* (low dose [LD]). Group 5 received 300 mg/kg b.wt. of *CM* (high dose [HD]); Group 6 received 0.03 mg/kg b.wt. of tamsulosin plus 30 mg/kg b.wt. of *CM*. Group 7 received 0.03 mg/kg b.wt. plus 300 mg/kg b.wt. of *CM*. Tamsulosin and *CM* were administered by oral gavage for 28 days. Prostate-specific antigen (PSA) levels, renal and liver function tests, and histology were assessed.

**Results:** PSA decreased after treatment with LD *CM* (0.44 ± 0.03 ng/mL) and tamsulosin (0.43 ± 0.04 ng/mL) combined, compared with the control group (0.63 ± 0.03 ng/mL) (*p* < 0.006). Prostate gland/accessory organ weights were as follows: tamsulosin < *CM* LD < *CM* HD/T < *CM* LD/T < *CM* HD < model. In the *CM* LD/T group, the acini appeared empty and the acini fluid contained fatty droplets with a slender outer boundary that had very little active mucous surface. At a higher dose, *CM* HD/T caused a reduction in the sizes and shapes of active acini with most being empty and having little active mucous surfaces.

**Conclusion:** The results suggest that the combination of *C. membranaceus* and tamsulosin does not provide additional therapeutic benefits for treating BPH. Relative organ weights provide a better evaluation metric than total organ weights.

## 1. Introduction

Benign prostatic hyperplasia (BPH) is a condition characterized by the proliferation of epithelial and smooth muscle cells in the transition zone of the prostate gland [1]. Thus, BPH lesions are based on clinical, gross, and histopathological studies of the prostate and other accessory glands of the urogenital system. Clinically, this condition manifests as lower urinary tract symptoms (LUTSs), which include urgency to urinate, nocturnal urination, weak urine stream, and difficulty in fully emptying the bladder [2]. Although BPH and LUTS may be viewed as distinct conditions, BPH often leads to LUTS [3]. The condition, BPH, is primarily age-related and affects up to 80% of men over the age of 80 [4]. However, microscopic signs of BPH may develop in men as early as in their 20s and 30s. It is rare for men in their 20s to experience significant symptoms of BPH or an enlarged prostate. Typically, urinary symptoms in men aged 20–30 years have other underlying causes. Symptoms may be caused by an enlarged prostate, but often it is swollen from infection rather than large from growth. Symptomatic BPH is not very common in men who are aged 30–39, but a significant enough number of men start to show symptoms of an enlarged prostate at this age. Typically, symptoms are mild, but occasionally symptoms are moderate or severe enough that they require treatment. Metabolic risk factors such as diabetes, obesity, and hypertension have contributed to the increasing incidence of BPH [5].

Another age-related factor is the decline in testosterone levels, which decreases by 2%–3% annually in men [6]. Age and testosterone levels have long been related to BPH [7]. However, the exact mechanism remains unclear. A sharp decline in testosterone around the age of 40, coinciding with the emergence of BPH, has led researchers to consider the role of other hormones such as estrogen [8, 9]. Estrogen signals trigger the amount of androgen receptors in the prostate gland even in the presence of declining testosterone levels [10]. The resultant effect of these events is the proliferation of mesenchymal stem cells (MSCs) that play an important role in the development of human prostate cancer (PCa). However, the role of MSCs in the transformation of androgen-dependent human PCa cells into an androgen-independent phenomenon has been poorly understood. The accumulation of these mesenchymal-like cells, originating from the prostate epithelium rather than the stroma, is believed to underlie BPH [11].

In the management of BPH, medications developed consider the mechanistic pathways that interfere with the hormones or the receptors on the prostate. To mitigate the symptoms of BPH, five categories of drugs are generally prescribed including phosphodiesterase type 5 inhibitors, beta-3-adrenergic agonists, anticholinergic agents, alpha-reductase inhibitors, and 5-alpha-reductase inhibitors. The first three target the reaction of the smooth muscles of the bladder and surrounding vasculature. The other two categories, 5-alpha-reductase inhibitors, prevent the conversion of testosterone to dihydrotestosterone (DHT). The 5-alpha-reductase inhibitors, such as finasteride and dutasteride, are commonly used to manage prostate conditions. Another class of medications, alpha-1 blockers—including tamsulosin, terazosin, alfuzosin, doxazosin, and silodosin—plays a dual role in regulating prostate growth. They promote apoptosis and reduce prostate smooth muscle contraction by blocking smooth muscle receptors. This mechanism helps decrease resistance to urine flow, improving urinary symptoms.

Tamsulosin is one of the most widely used alpha-1 blockers. Recent research has used supercritical antisolvent (SAS) methods to produce micro- and nanoparticles of tamsulosin, improving its solubility, reducing required doses, and minimizing side effects [12]. However, the drug has some undesirable complications, including reduced libido and erectile dysfunction.

As a result, there is a growing global interest in natural phytomedicines, which are perceived to be safer and have fewer side effects. A systematic review of available herbal treatments for BPH found that *Serenoa repens* (saw palmetto) representing 54% is the most studied and widely used herbal remedy, followed by *Urtica dioica*, *Cucurbita pepo*, *Pygeum africanum*, and *Linum usitatissimum* [13]. Several studies have reported that *Serenoa repens*, either used alone or in combination with other treatments, improves BPH symptoms without significantly affecting prostate size.

*S. repens* is commonly known as saw palmetto in the southern regions of North America [14]. Of the 15 studies on *S. repens* for BPH management in isolation or as a combination, 13 reported improvements in the International Prostate Symptoms Score (IPSS), with 11 showing statistical significance. In addition, 10 studies had reported improvement in prostate-specific antigen (PSA), while 4 demonstrated improvement in the quality of life (QoL) [13]. The improvement in mild-to-moderate LUTS with its use is comparable to finasteride, a 5-alpha-reductase inhibitor [15]. On the contrary, later updates suggested that *S. repens* did not significantly improve LUTS nor reduce the prostate size [16]. Notably, extensive studies have been conducted on *S. repens* in combination with tamsulosin.

In a meta-analysis, six articles have been identified as having comparative studies on *α*-blockers and *S. repens* combination use. It was concluded that patients with prostatic inflammatory status (PIS) might benefit from this combination therapy and could be added to the standard first-line treatment with alpha blockers [17]. A 6-month trial combining 0.4 mg per kg of tamsulosin with 320 mg per kg of hexane extract from *Serenoa repens* demonstrated significant symptom relief and improved QoL in patients with moderate-to-severe LUTSs associated with BPH [18].

In an animal study, D-004, an oil extract from *royal palm,* was used in combination with tamsulosin to treat urodynamic changes in phenylephrine BPH-induced rats (D-004 400 mg/kg *royal palm* + tamsulosin 0.05 mg/kg). In that study, the rat's urine volume voided per micturition was measured. It was found that there was a reduction in urine volume voided, which was indeed significant and occurred in a dose-dependent manner. The combined therapy yielded better results than the corresponding monotherapy [19].

In West Africa, one common plant used for BPH treatment is *C. membranaceus*. Several studies have suggested some level of efficacy in both animal models [20, 21] and human studies [22]. In the human study, there was a 40.8% reduction in PSA and a 46.6% reduction in prostate volume over 3 months. Several putative pathways for the action of *C. membranaceus* have been reported: the antioxidant pathway [23], the apoptotic pathway [21], the sphingolipid pathway [23], and the 5-alpha-reductase pathway [24]. Calcium magnesium imbalance and its correction through the use of *C. membranaceus* have also been reported [22]. Eleven products on the Ghanaian market have been identified to contain the generic product *C. membranaceus,* with at least four of them being FDA-approved [25]. It is of concern whether these natural products are being used in combination with some allopathic drugs, although such has been suggested in communications. A recently published paper focused on the combination of *C. membranaceus* with finasteride (5-*α* reductase inhibitor). The combination was reported not to have any overt therapeutic advantage, even though there was good tolerability [26]. However, unlike some research works combining some medicinal plants such as *S. repens* with tamsulosin, no study has considered the combination of *C. membranaceus* (a 5-*α* reductase inhibitor) and tamsulosin (an −1 blocker) for efficacy or safety. Considering that no extant literature has explored the combined use of *C. membranaceus* and tamsulosin for BPH treatment in a clinical study, this current investigation presents novelty as it highlights the potential benefits and risks of combined therapy. It further provides important safety data on this combination, a crucial step in advancing the field of integrative medicine, particularly in regions such as Ghana (Africa) where herbal medicine is widely used. Additionally, the research contributes new knowledge regarding the limitations of combining herbal and allopathic treatments, challenging the assumption that such combinations always yield better outcomes.

The present study aimed at determining the effect of *C. membranaceus* and tamsulosin combination, in the management of BPH.

## 2. Materials and Methods

### 2.1. Plant Collection, Extraction, and Identification

The roots of *Croton membranaceus* (CM) were collected from Mampong Akwapim (latitude 5.9246 N and longitude 0.2332 W). The plants were harvested from wild populations in the region. Plant collection was conducted under the supervision of the Centre for Plant Medicine Research, with adherence to local and international ethical guidelines for the sustainable collection of medicinal plants. Additionally, the voucher specimen information (CSRP 2110) was recorded.

The roots were washed to remove sand and debris and then air-dried under subdued light for 1 month. Once dried, the roots were milled into a fine powder, which was stored in zip-lock bags at room temperature.

To prepare the extract, 1 kg of the powder was mixed with 4 L of distilled water and left to macerate for 24 h. The volume of 4 L of water per kilogram of plant material was selected based on empirical optimization to ensure adequate solvent penetration and maximize extraction efficiency. The maceration process was conducted under aseptic conditions using sterile containers and distilled water to prevent microbial contamination. A decoction at 100°C for 1 hour ensured the elimination of any potential microbes. The processed extract was immediately freeze-dried to preserve its integrity and prevent microbial growth. The freeze-dried product was then stored at 2–8°C yielding 2.5% of the original mass. The freeze-dried extract was stored at 2°C–8°C until further use. The extraction was performed following the protocol of previous work [20].

#### 2.1.1. Details on Lyophilizer Model

Model: Labconco FreeZone 4.5 L Benchtop Freeze Dryer.

#### 2.1.2. Specifications

Type: Benchtop model.

Condenser Temperature: −50°C (−58°F).

Vacuum Pump Requirement: Minimum 98 L/min (3.5 CFM) displacement.

Capacity: 4.5 L per run.

Material Compatibility: Suitable for aqueous and light organic solvent samples.

User Interface: Digital controls with temperature and pressure display.

#### 2.1.3. Protocol to Avoid Potential Contamination

##### 2.1.3.1. Controlled Environment for Drying

The plant roots were dried in a well-ventilated room maintained at controlled temperature (25°C–30°C) and humidity (< 50%) to prevent microbial growth.

##### 2.1.3.2. Sterilization of Equipment and Area

The drying racks and surrounding area were sterilized before use to maintain hygiene.

##### 2.1.3.3. Drying Under Subdued Light

The drying process was carried out under subdued lighting to reduce microbial activity and preserve phytochemical integrity.

##### 2.1.3.4. Regular Monitoring

Periodic sampling of the drying material was conducted to check for signs of microbial contamination.

##### 2.1.3.5. Postdrying Sterilization

Postdrying, the plant material was sterilized using ethanol to eliminate potential contaminants.

##### 2.1.3.6. Packaging in Airtight Containers

The dried plant material was immediately sealed in sterile, airtight containers for storage.

#### 2.1.4. Development of BPH Model Using Sprague Dawley Rats

Ethics approval for the study was granted by the Kwame Nkrumah University of Science and Technology, with ethics number CHRPE/AP/157/23. Thirty-five pathogen-free Sprague Dawley rats weighing between 200 and 250 g were obtained from the Department of Animal Experimentation at the Noguchi Memorial Institute for Medical Research.

The rats were housed under a 12-h light/dark cycle, with temperatures maintained at 22 ± 3°C and relative humidity at 40%–45%. They were given free access to sterilized water and a standard chow diet. After a 7-day acclimatization period, 28 rats underwent orchidectomy (castration) to induce BPH. After the surgery, the rats were allowed to recover for 7 days. Postrecovery, the rats were randomized into six groups and injected with 5 mg/kg body weight of testosterone propionate (CAS Number 57-85-2; Auckland, New Zealand) subcutaneously for 28 days to induce BPH, following the protocol [27]. The 35 rats were divided into the following seven groups, each with five rats:

• Control Group: Rats received saline.
• Model Group: Untreated BPH-induced rats.
• Tamsulosin Group: Rats treated with 0.03 mg/kg body weight of tamsulosin (CAS Number 106463–17–6; Exeter Pharmaceuticals, UK).
• Low-Dose *C. membranaceus* (CM LD) Group: Rats treated with 30 mg/kg body weight of *C. membranaceus.*
• High-Dose *C. membranaceus* (CM HD) Group: Rats treated with 300 mg/kg body weight of *C. membranaceus.*
• Tamsulosin + CM LD Group: Rats treated with a combination of 0.03 mg/kg body weight of tamsulosin and 30 mg/kg body weight of *C. membranaceus.*
• Tamsulosin + CM HD Group: Rats treated with a combination of 0.03 mg/kg body weight of tamsulosin and 300 mg/kg body weight of *C. membranaceus.*

Tamsulosin and *C. membranaceus* were administered orally via gavage for 28 days. At the end of the experiment, the rats were weighed and then anesthetized using ketamine (CAS Number 6740-88-1; Hameln, Germany). Blood samples (5 mL) were collected via cardiac puncture for hematological and biochemical analyses. Rats were euthanized using isoflurane, and internal organs (prostate, liver, kidneys, heart, spleen, and lungs) were harvested for further examination.

### 2.2. Biochemical and Hematological Assays

Blood collected in EDTA tubes was used for full blood count (FBC) analysis, while blood in gel separator tubes was used for biochemical assays, including liver and kidney function tests. Testosterone and other biochemical parameters were analyzed using a Beckman Coulter AU480 Chemistry Analyzer (New York, USA). FBC was analyzed using an ACT5 Beckman Coulter instrument (New York, USA).

PSA levels were determined using an ELISA kit specific for rats (Elabscience, Wuhan, China). The test was conducted according to the manufacturer's protocol, with optical density read at 450 nm on a BioSystems ELISA plate reader (Madrid, Spain).

### 2.3. Histopathological Analysis

Harvested organs were weighed and fixed in 10% buffered neutral formalin for histological analysis. Tissue samples from the prostate and seminal vesicles were processed and stained with hematoxylin and eosin, following standard protocols [28]. Sections were cut at 5 μm thickness and examined microscopically using an Olympus microscope (Tokyo, Japan).

### 2.4. Dose Selection

The dose of 0.03 mg/kg b.wt. for tamsulosin was chosen based on previously published studies and allometric scaling from the human therapeutic dose. This dose is consistent with protocols reported in preclinical BPH models, ensuring efficacy while minimizing adverse effects [29]. Preliminary testing also confirmed the safety and tolerability of this dose in Sprague Dawley rats. The 300 mg/kg high dose was chosen based on previous studies where rats received CM doses up to 5000 mg/kg without any signs of toxicity or fatal effects [29]. These studies also demonstrated efficacy in preclinical BPH models, supporting its use as a therapeutic dose.

### 2.5. Ethical Disposal of Animal Carcasses

To ensure compliance with ethical standards, the disposal of animal carcasses was conducted following established institutional and international guidelines.

#### 2.5.1. Adherence to Ethical Guidelines

Animal carcasses were handled and disposed of in accordance with the principles outlined in the *Guide for the Care and Use of Laboratory Animals* and the ethical standards of the Kwame Nkrumah University of Science and Technology.

The protocol was reviewed and approved by the Institutional Animal Care and Use Committee (IACUC) under Approval Number CHRPE/AP/157/23.

#### 2.5.2. Disposal Method

After euthanasia, carcasses were placed in sealed biohazard bags to prevent contamination and transported to an approved incineration facility.

The incineration process ensured the complete and environmentally safe disposal of biological material.

#### 2.5.3. Environmental Safety Measures

All disposal procedures were designed to minimize environmental risks and comply with local biohazard waste management regulations. The incineration facility used adhered to safety and environmental guidelines, ensuring no release of harmful substances during the process.

#### 2.5.4. Oversight and Monitoring

The disposal was supervised by trained technical staff to ensure compliance with biohazard handling protocols and ethical standards. Records of carcass disposal were maintained as part of the study's documentation for transparency and accountability. This protocol underscores the commitment to maintaining high ethical standards in research involving animal subjects while ensuring safe and environmentally responsible disposal methods.

### 2.6. Data Analysis

Data were analyzed using SPSS version 26. Descriptive statistics were reported as mean ± standard error of the mean (SEM). Analysis of variance (ANOVA) was performed to assess differences between groups, followed by Sidak's post hoc multiple comparisons test to determine significant differences between treatments. A *p* value < 0.05 was considered statistically significant.

## 3. Results

### 3.1. Organ Weights

The absolute weights of the organs showed that the model group (untreated BPH) had the highest overall organ weights, especially for the prostate and seminal vesicles. All treatment groups, including those treated with tamsulosin and *C. membranaceus*, exhibited reduced organ weights compared with the model group, but still showed significant increases relative to the control group (*p* < 0.05) (Table 1). For the heart, the model group and *C. membranaceus*–treated groups showed significant differences in weight compared with the control group (*p* < 0.05). The highest lung weight was recorded in the CM HD group, with statistical significance when compared to the control group (*p*=0.03). Similarly, the prostate and seminal vesicles exhibited increased weights compared with the control, with the model group showing the highest values. However, other organs such as the liver, spleen, and kidneys did not show significant differences across groups (Table 2).

### 3.2. Hematological and Biochemical Parameters

The FBC did not show any level of difference among the various groups (Table 3). The biochemical data included renal function tests (urea, creatinine, Na, K, and Cl). This showed significant differences in creatinine. The tamsulosin group and the LD *CM* were significantly lower compared with the model group (*p*=0.05). No differences, however, were recorded with the other parameters. For the liver function tests (ALT, AST, ALP, TBIL, DBIL, total protein [TP], and ALB), only TP was significantly lower in the tamsulosin and the combined tamsulosin and *CM* group (Table 4).  Table 5 shows that testosterone was highly elevated in all the test groups compared with those of the control group (*p*=0.05). PSA value was the highest in the control group. However, the LD of *CM* plus tamsulosin had significantly lower PSA values than the control group.

### 3.3. Histopathological Findings

**Figure 1:**
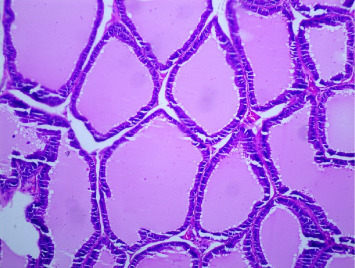
Section through the prostate from a rat that received normal saline showed a normal structure. The acini are lined with epithelium and surrounded by loose, fibrous connective tissue containing smooth muscle fibers and blood vessels. The epithelial cells of the acinar lining are cylindrical, prismatic, and/or cuboidal with a basal nucleus. H&E × 100.

**Figure 2:**
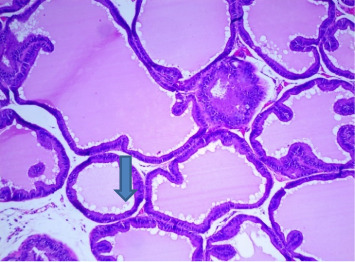
A section through a prostate from a rat injected with testosterone to induce hyperplasia of the prostate after being castrated. Microscopically, hyperplastic glands of variable sizes with papillary folding were found in the acini. Gland surfaces were lined with double-layered epithelium inner columnar and outer cuboidal. The fibromuscular stroma showed a typical characteristic of high epithelial folds in the distal region of the gland. Some areas showed apparent epithelial hyperplasia. The empty vacuoles were demonstrated (arrowed) (H&E × 100).

**Figure 3:**
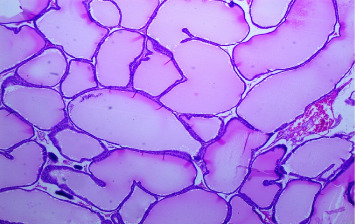
A section through a prostate from a rat experimentally induced to develop BPH and later treated with *Cm* (30 mg/kg b.wt.) did not have enough apoptotic areas, but multiple layers (hyperplasia) of the acinar epithelia were noticed (H&E × 100).

**Figure 4:**
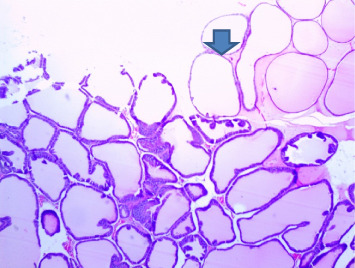
Section through a prostate from a rat that was experimentally produced BPH and later treated with *Cm* (300 mg/kg b.wt.). *Note:* Acinar fluid was found to be reduced, and some of them were empty (arrowed). There were a few apoptotic cells on the epithelial surface of the acini. Interstitial stroma showed infiltrations of mononuclear cells (H&E × 100).

**Figure 5:**
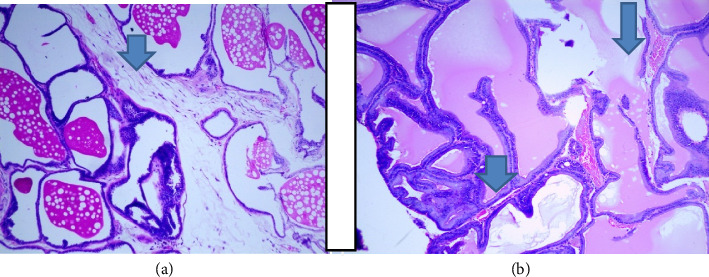
Section through a prostate from a rat that was experimentally produced BPH and later treated with tamsulosin (0.03 mg/kg b.wt.) as a positive control. (a) Empty acini and fat bodies that could have been recognized in the condensed acinar fluid. There was extreme fibrosis and infiltration of immature fibroblasts that appeared as purple thread with centrally placed nuclei. (b) Hyperplasia on the acinar surface which almost collapsed the inner cavity (arrowed). However, many showed drying of the acinar colloid (arrowed) (H&E × 100).

**Figure 6:**
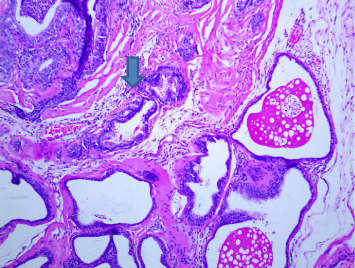
Section through a prostate from a rat that was experimentally produced BPH and later treated with tamsulosin (0.03 mg/kg b.wt.) and *Cm* (30 mg/kg b.wt.). *Note:* Acini appeared empty, and the acinar fluid contained fatty droplets. The stromal tissues showed fibrosis and contracted (arrowed) (H&E × 100).

**Figure 7:**
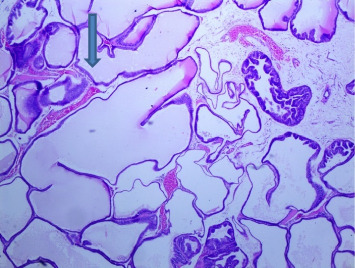
Section through a prostate from a rat that was experimentally produced BPH and later treated with tamsulosin (0.03 mg/kg b.wt.) and *Cm* (300 mg/kg b.wt.) (H&E × 100). *Note:* There was a tendency to reduce the size and shape of the active acini. Most of the acini were left empty with a slender outer boundary, which had very little active mucous surface (arrowed) (H&E × 100).

There were no gross changes in the prostate and other accessory organs related to the genitourinary system of rats that received experimental preparations and parenteral administration of drugs mentioned previously. Microscopically, the control group showed a normal prostate architecture with normal epithelial lining and the acini with smooth muscle fibers and blood vessels (Figure 1). The BPH-induced untreated group demonstrated hyperplastic glands with papillary folding into the acini representing an apparent epithelial hyperplasia (Figure 2). Figures 3 and 4 demonstrate the reduction in the epithelium and stroma after *CM* administration at lower doses (LDs) and higher doses (HDs), respectively. However, at the level of HD, representative prostate sections from the rats showed infiltrations of mononuclear cells (Figure 4) in the stroma. With tamsulosin, there was infiltration, and the acini were found to be empty, and however, fat bodies appeared to be condensed within the acinar fluid. There were infiltrations of immature fibroblasts (Figure 5(a)) in the stromal linings. Hyperplasia on the acini surface had almost disappeared (Figure 5(b)). Effects of the combination of *CM* (LD) and tamsulosin on the prostate gland are shown in Figures 6 and 7. Stromal tissues showed fibrosis and thus were contracted. Similarly, alterations were recorded at the level of HDs of *CM* in conjunction with tamsulosin. The acini appeared to be empty with scanty active mucus-lining cells (Figure 5).

### 3.4. Lesions in the Seminal Vesicles

**Figure 8:**
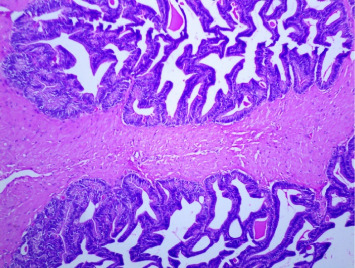
Section through a seminal vesicle from a control rat. *Note:* The smooth muscle cells in the tunica muscularis were strongly stained. The nuclear staining intensity of these cells was similar to luminal epithelial cells. The staining intensity in luminal cells was stronger than in basal cells (H&E × 100).

**Figure 9:**
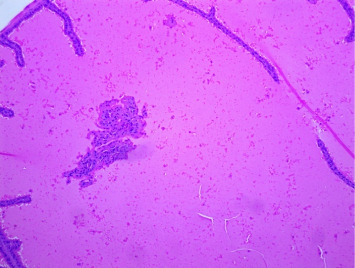
Section through a seminal vesicle of a rat. It was injected with testosterone to induce hyperplasia of the prostate after being castrated. *Note:* Clump of mononuclear cells in the vesicular fluid (H&E × 100).

**Figure 10:**
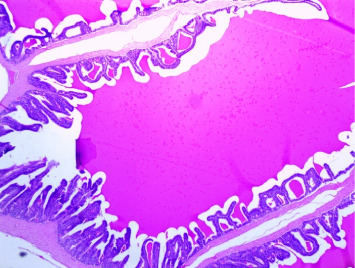
Section through a seminal vesicle from a rat that was experimentally produced BPH and later treated with *Cm* (30 mg/kg b.wt.). *Note:* Papillary projections in the acini were found to be increased. There were no significant alterations and infiltrations in the stroma or acini (H&E × 100).

**Figure 11:**
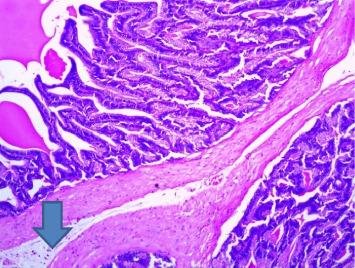
Section through a seminal vesicle from a rat that was experimentally produced BPH and later treated with *Cm* (300 mg/kg b.wt.). *Note:* Seminal fluid appeared to be reduced and condensed. The obvious decrease in fibrous tissue was found in the seminal vesicles, and inflammatory cell infiltration was also found in the stroma. Inflammatory cells infiltrated in the stroma of the seminal vesicle (arrowed) (H&E × 100).

**Figure 12:**
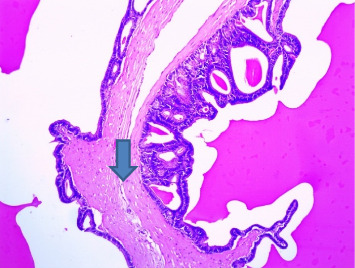
Section through a seminal vesicle from a rat that was experimentally produced BPH and later treated with tamsulosin (0.03 mg/kg b.wt.) and *Cm* (30 mg/kg b.wt.). *Note:* Acini appeared smaller and the acinar mucous surfaces. The stromal tissues showed fibrosis and contracted due to loss of active acini (arrowed). The papillary projections were found to be slender (arrowed) (H&E × 100).

**Figure 13:**
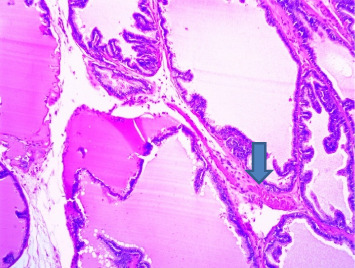
Section through a seminal vesicle from a rat that was experimentally produced BPH and later treated with tamsulosin (0.03 mg/kg b.wt.) and *Cm* (300 mg/kg b.wt.) (H&E × 100). Papillary projections in the acini became thinner with fat bubbles around. Infiltrations of cellular elements were found in the stroma (arrowed) (H&E × 100).

**Figure 14:**
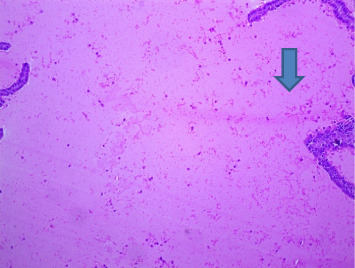
Section through a prostate from a rat that was experimentally produced BPH and later treated with tamsulosin (0.03 mg/kg b.wt.) as a positive control. Atrophy of the papillary projections was noticed on the mucous surface (H&E × 100).

Figure 8 shows the normal architecture of the seminal vesicle. The model group showed a clump of mononuclear cells in the vesicular fluid (Figure 9). At a LD of *CM*, papillary projections appeared to have increased (Figure 10). However, with the high dose of *CM*, there was a decrease in proliferations of fibrous connective tissues (Figure 11). In the combination groups (tamsulosin and *C. membranaceus*), the acini appeared smaller, with contraction of the stromal tissue (Figure 12). The high-dose combination treatment led to thinner papillary projections with fat bubbles around the acini and further cellular infiltration in the stroma, indicative of ongoing tissue remodeling (Figure 13). The experimentally produced BPH model treated with tamsulosin (0.03 mg/kg b.wt.) showed atrophy of the papillary projections on the mucous surface (Figure 14).

## 4. Discussion

The current report describes some alterations that have been recorded from rat studies with *CM* and a patent drug, tamsulosin, that are used to control BPH. To validate the investigation, the plant extract was prepared and stored as recommended in [30] before use. It is a fact that the prostate gland is both an endocrine and exocrine organ. As an endocrine gland, it is engaged in the metabolism of testosterone into a more effective androgen. However, as an exocrine gland, it produces prostatic fluid, which is rich in proteins, enzymes, lipids, minerals, etc. This prostatic fluid provides spermatozoa with the right acidity for its mobility and high levels of zinc for its viability.

In this study, the weights of the prostate gland and other accessory organs (seminal vesicle) were of increasing order: tamsulosin < *CM* LD < *CM* HD/T < *CM* LD/T < *CM* HD < model. As an *α*-1 blocker, tamsulosin is known to improve urine flow by relaxing the muscles in the bladder and prostate.

In terms of relative organ weight, tamsulosin was found to give lower weight followed by *CM* low dose. Either drug is likely active in providing relief of the symptoms of BPH. However, 5-*α* reductase inhibitors (as in the case of *CM*) are slow-acting although they eventually lead to a reduction in the size of the prostate gland and improvement in bladder outflow [30].

In this study, the combination therapies showed higher prostate/accessory organ weight than those of *CM* LD and tamsulosin. Hachimijiogan and Ryutanshakanto are Chinese herbal preparations for BPH. In a combination therapy study that was double-blind, tamsulosin/Hachimijiogan and tamsulosin/Ryutanshakanto were used for BPH patients for 12 weeks. Both combinations increased prostate volumes. However, IPSS, QoL, and uroflowmetry parameters improved significantly [31]. Thus, weight alone may not be an indicator of improvement.

In another study involving tamsulosin and *Serenoa repens*, a 5-*α* reductase inhibitor similar to *CM*, Qmax, and IPSS failed to show any improvement. Therefore, the combination therapy did not offer any additional therapeutic benefit per se [32]. For PSA, *CM* LD and *CM* LD/T were lower than all other groups and significant when compared to those of the control group. However, these were not significant when compared to those values obtained from the model group. In a study involving tamsulosin and two Chinese herbal drugs, PSA levels also did not increase as seen in that study despite clinical improvement [31]. In another study conducted with 279 men between 2008 and 2010 in Korea, tamsulosin or *Serena repens* or the combination was administered. Both prostate volume and PSA did not show any significant difference. These authors believed that the independent use of tamsulosin and *Serena repens* was effective without any demonstration of superiority. Although the combination was well tolerated, it did not offer any additional benefit [33]. Permixon, a product of *Serena repens,* showed a slight change in prostate volume with PSA unchanged, when it was administered in comparison with tamsulosin alone for a year among 542 patients [34]. Again, PSA levels did not correlate with the prostate volume.

Histopathologically, the acini of the prostate are lined with secretory cells or prostasomes that empty into ducts within the prostate [35]. Comparatively, at *CM* LD, no empty acini were reported. At an HD of *CM*, a few empty acini were reported with reduced numbers as well. However, in the *CM* LD/T the acini appeared empty and the acini fluid contained fatty droplets with slender outer boundaries that had very little active mucous surface. Boundary cells have been identified as epithelial cells residing in a different environment compared with the basal cells in the acinar regions; they are in the ridges separating the acini [35].

At a HD, *CM* HD/T caused a reduction in the sizes and shapes of active acini with most of them empty and having little active mucous surfaces. These events are characteristic of hyperplasia. Of greater implication is the fact that the combination treatment led to prostate atrophy: a reduction in the size and number of acini. Prostate atrophy is a condition in which the prostate gland's tissue shrinks, often as a result of aging. The phenomena can also be associated with hormonal changes.

For tamsulosin alone, acini were empty and fat bodies could be recognized in the condensed acinar fluid. There was extreme fibrosis and infiltration of immature fibroblasts that appeared as purple thread with centrally placed nuclei. The primary function of fibroblasts is to maintain the structural integrity of connective tissues [35]. Hyperplasia on the acinar surface had almost collapsed the inner cavity. Thus, tamsulosin on its own effectively demonstrated antihyperplastic activities.

It appears that the combination, both at the LDs and HDs, affected the exocrine function of the prostate, leading to the senescence of secretory cells. Stromal tissues showed fibrosis and contracted.

For the seminal vesicles, acini appeared smaller in the LD combination, and at the HD combination, acini became thinner with bubbles around and with infiltration of cellular elements in the stroma. Hormonal imbalances, such as changes in androgen (e.g., testosterone) levels, can influence the structure and function of the seminal vesicles. Cellular infiltration in the stroma may be related to hormonal changes affecting the organ. When there is a depletion of androgenic hormones, atrophic effects on the seminal vesicle are profound. Estrogen is said to decrease glandular epithelium volume while increasing the prominence of the fibromuscular stroma in the seminal vesicle [36].

With the absolute organ weights, the lungs and heart increased. Nonetheless, *CM* LD was lower than tamsulosin for the heart and vice versa for the lungs. None was significant compared with the model group. A dose-dependent increase was similarly observed in another 90-day study with SD rats. However, increases were not statistically significant [24].

Creatinine levels were significantly lower in tamsulosin and *CM* LD than in the corresponding control group, and these were much lower than both LD and HD combinations. This may suggest that *CM* LD and tamsulosin are nephroprotective. Two studies by [20, 24] using *CM* had creatinine levels literally unchanged in subchronic toxicity studies (90 days) with SD rats. In a human study on the management of BPH with *CM*, creatinine remained virtually unchanged at 20 mg t.i.d. for 90 days [30].

TP was significantly lower in the tamsulosin and *CM* HD/T groups. In an acute toxicity study using a high dose of 3000 mg/kg b.wt., total protein was not affected [26, 30]. Similarly, in the chronic toxicity study, TP remained unchanged [24]. All other liver parameters did not show significant differences, likewise the hematology parameters.

The study demonstrated that both *CM* and tamsulosin were well tolerated, with no significant changes in liver or kidney function markers across the treatment groups. However, the lack of functional assessments, such as urinary flow rates, limits the conclusions that can be drawn regarding the overall clinical benefit of these treatments. Overall, the results suggest that the coadministration of *CM* and tamsulosin may not be a beneficial strategy for BPH management. Monotherapy with either *CM* or tamsulosin could be considered more effective and safer based on the parameters assessed in this study. Further research is needed to explore longer-term effects, functional outcomes, and alternative dosing strategies to optimize treatment for BPH, particularly when considering combination therapies.

### 4.1. Significance of Relative Weights

Relative organ weights offer a more accurate evaluation metric for treatment effects than total organ weights, especially in BPH models.

### 4.2. Combination Therapy

While CM and tamsulosin independently showed some therapeutic benefits, their combination did not yield additional advantages. Histological findings of fibrosis and stromal contraction warrant further investigation.

## 5. Conclusion

The present study evaluated the safety and potential therapeutic benefits of combining CM, a phytomedicine, with tamsulosin, an allopathic alpha-1 blocker, in a rat model of BPH. The findings indicate that while both CM and tamsulosin are effective in reducing prostate weight and PSA levels, their combined use does not provide any additional therapeutic advantage. In fact, the combination of CM and tamsulosin led to increased stromal fibrosis and atrophy of prostate acini, raising concerns about possible adverse tissue remodeling effects. Further studies are required to optimize dosing and evaluate long-term outcomes.

### 5.1. Limitations of the Study

This study is not without limitations. The duration of the study was relatively short (28 days), which may not have been sufficient to observe the long-term effects of the treatments, particularly in a chronic condition like BPH. Moreover, the study focused on basic biochemical and histological parameters but did not include functional tests, such as measurements of urinary flow rate, which are crucial for assessing the therapeutic efficacy of tamsulosin and its combination with *CM.*

Additionally, while this study focused on the prostate and accessory organs, it did not thoroughly explore the potential systemic effects of *CM* and tamsulosin, particularly their interactions in metabolic and hormonal pathways that may contribute to BPH. Further studies are needed to understand the underlying mechanisms and to assess the long-term safety and efficacy of these treatments.

## Figures and Tables

**Table 1:** Mean organ weight of rats on various drug regimens.

**Organ weight (g)**		**Model**	**30 mg/kg b.wt. *Cm***	**300 mg/kg b.wt. *Cm***	**30 mg/kg b.wt. *Cm*/tamsulosin**	**300 mg/kg b.wt. *Cm*/tamsulosin**	**Tamsulosin**	**p** **value**

Heart	0.76 ± 0.02	0.98 ± 0.05⁣^∗^	0.88 ± 0.06	0.94 ± 0.05⁣^∗^	0.94 ± 0.04⁣^∗^	0.92 ± 0.04	0.83 ± 0.03	**0.000**
Lungs	1.42 ± 0.20	1.98 ± 0.11	1.7 ± 0.04	2.38 ± 0.22⁣^∗^	1.80 ± 0.14	1.60 ± 0.11	1.90 ± 0.13	**0.037**
Liver	6.40 ± 1.08	8.43 ± 0.38	6.62 ± 0.36	7.16 ± 0.60	8.64 ± 0.49	7.34 ± 0.31	7.25 ± 0.29	0.092
Spleen	2.12 ± 0.86	1.0 ± 0.11	0.92 ± 0.05	1.04 ± 0.07	1.04 ± 0.05	0.86 ± 0.02	1.08 ± 0.08	0.215
Brain	1.44 ± 0.19	1.75 ± 0.09	1.62 ± 0.09	1.76 ± 0.09	1.78 ± 0.04	1.56 ± 0.14	1.68 ± 0.05	0.329
Kidney	0.84 ± 0.02	1.08 ± 0.09	0.94 ± 0.02	1.00 ± 0.09	1.06 ± 0.06	0.90 ± 0.03	0.98 ± 0.09	0.014
Seminal vesicle and prostate	1.68 ± 0.06	4.9 ± 0.21⁣^∗^	3.42 ± 0.42⁣^∗^	3.70 ± 0.61⁣^∗^	3.68 ± 0.09⁣^∗^	3.58 ± 0.36⁣^∗^	3.35 ± 0.40	**0.000**

*Note:* Data presented as mean ± SEM. The bold values indicate statistical significance.

⁣^∗^*p* < 0.05 compared with the control group.

**Table 2:** Relative mean organ weight of rats on various drug regimens.

Organs (Wt = ×10^−3^)	Control	Model	30 mg/kg b.wt. *Cm*	300 mg/kg b.wt. *Cm*	30 mg/kg b.wt. *Cm*/tamsulosin	300 mg/kg b.wt. *Cm*/tamsulosin	Tamsulosin
Heart	3.16^−^±0.13	3.51 ± 0.18	3.41 ± 0.16	3.47 ± 0.15	3.31 ± 0.13	3.47 ± 0.23	3.20 ± 0.15
Lungs	5.84^−^±0.74	7.10 ± 0.45	6.64 ± 0.39	8.73 ± 0.50^†^	6.67 ± 0.69	6.00 ± 0.45	7.37 ± 0.55
Liver	26.20 ± 4.10	30.20 ± 1.10	20.57 ± 1.06	20.64 ± 1.95	30.50 ± 1.20	20.75 ± 1.06	28.10 ± 1.18
Spleen	9.12 ± 0.39	3.59 ± 0370	3.56 ± 0.13	3.84 ± 0.24	3.78 ± 0.33	3.18 ± 0.16	4.18 ± 0.37
Brain	5.91 ± 0.72	6.28 ± 0.20	6.28 ± 0.31	6.50 ± 0.29	6.38 ± 0.38	5.85 ± 0.57	6.49 ± 0.23
Kidney	3.49 ± 0.11	3.86 ± 0.29	3.66 ± 0.20	3.32 ± 0.16	3.86 ± 0.29	3.38 ± 0.16	3.83 ± 0.49
Seminal vesicle and prostate	6.96 ± 0.15	13.6 ± 4.19^†^	13.2 ± 1.47^†^	13.5 ± 1.99^†^	13.30 ± 0.97^†^	13.4 ± 1.28^†^	12.9 ± 1.40

*Note:* Data presented as mean ± SEM.

^†^ *p* < 0.05 compared with the control group.

**Table 3:** Mean hematological parameters of rats on various drug regimens.

FBC	Negative control	Model	30 mg/kg b.wt. *Cm*	300 mg/kg b.wt. *Cm*	30 mg/kg b.wt. *Cm*/tamsulosin	300 mg/kg b.wt. *Cm/*tamsulosin	Tamsulosin	*p* value
WBC (10^9^/L)	4.96 ± 0.90	9.43 ± 0.75	9.98 ± 1.86	8.32 ± 2.06	10.18 ± 2.54	12.26 ± 2.27	13.08 ± 6.25	0.458
RBC (pL)	8.52 ± 0.72	8.82 ± 0.36	6.16 ± 1.43	6.83 ± 1.38	7.86 ± 0.91	9.14 ± 0.42	7.478 ± 0.97	0.376
HBG (g/dL)	14.32 ± 1.09	14.83 ± 0.21	10.22 ± 2.23	10.76 ± 2.01	13.45 ± 1.45	14.48 ± 0.51	11.55 ± 1.49	0.233
HCT (L/L)	0.45 ± 0.04	0.46 ± 0.01	0.31 ± 0.07	0.3374 ± 0.06	0.41 ± 0.05	0.45 ± 0.02	0.36 ± 0.05	0.244
MCV(FL)	52.4 ± 0.60	52 ± 1.15	52.17 ± 1.42	50.8 ± 1.66	52 ± 0.41	49.2 ± 0.58	48.5 ± 1.04	0.180
MCH (pg)	16.88 ± 0.22	16.85 ± 0.62	13.93 ± 2.8	16.32 ± 0.73	17.25 ± 0.55	15.84 ± 0.25	15.48 ± 0.53	0.718
MCHC (g/dL)	32.20 ± 0.54	32.53 ± 0.83	27.32 ± 5.47	32.18 ± 0.49	33.20 ± 0.99	32.28 ± 0.27	31.93 ± 0.45	0.771
RDW-SD	12.66 ± 0.43	13.13 ± 0.23	11.75 ± 0.67	10.90 ± 1	13.60 ± 0.31	11.40 ± 1.11	10.85 ± 0.86	0.095
PLT (10^9^/L)	691.80 ± 236.80	774.75 ± 211.93	314.33 ± 127.82	297.40 ± 142.30	476.25 ± 170.61	601.20 ± 221.86	336.50 ± 267.53	0.588
LYP (%)	72.04 ± 3.98	71.20 ± 2.51	66.62 ± 4.16	69.60 ± 8.14	67.58 ± 3.18	66.68 ± 5.71	69.3 ± 3.55	0.991
LYA (10^9^/L)	3.61 ± 0.71	6.72 ± 0.68	6.69 ± 1.42	5.23 ± 0.75	6.92 ± 1.91	7.76 ± 1.14	8.88 ± 4.18	0.557
NEA (10^9^/L)	1.11 ± 0.21	2.06 ± 0.21	2.50 ± 0.50	2.764 ± 1.54	2.25 ± 0.48	3.18 ± 1.0	3.03 ± 1.27	0.622
MOA (10^9^/L)	0.25 ± 0.1	0.33 ± 0.04	0.41 ± 0.09	0.19 ± 0.06	0.70 ± 0.30	1.08 ± 0.49	0.84 ± 0.71	0.387
EOA (10^9^/L)	0.04 ± 0.02	0.26 ± 0.12	0.29 ± 0.18	0.104 ± 0.04	0.25 ± 0.11	0.11 ± 0.03	0.21 ± 0.11	0.584
BAO (10^9^/L)	0.04 ± 0.01	0.05 ± 0.01	0.07 ± 0.02	0.044 ± 0.01	0.08 ± 0.02	0.1 ± 0.02	0.13 ± 0.09	0.581

*Note:* Data presented as mean ± SEM. HGB = hemoglobin; HCT = hematocrit; PLT = platelet; LYM % = lymphocyte percentage; LYP = lymphocyte; LYA = lymphocyte count; NEA = neutrophil count; MOA = monocyte count; EOA = eosinophil count; BAO = basophil count.

Abbreviations: MCH = mean corpuscular hemoglobin; MCHC = mean corpuscular hemoglobin concentration; MCV = mean corpuscular volume; MPV = mean platelet volume; RBC = red blood cell; RDW-CV = coefficient of variation in red cell distribution width; RDW-SD = standard deviation in red cell distribution width; WBC = white blood cell.

**Table 4:** Mean renal and liver function test parameters of rats on various drug regimens.

Parameter	Control	Model	30 mg/kg b.wt. *Cm*	300 mg/kg b.wt. *Cm*	30 mg/kg b.wt. *Cm*/tamsulosin	300 mg/kg b.wt. *Cm*/tamsulosin	Tamsulosin	*p* value
Renal function test								
UREA (mmol/L)	6.16 ± 0.56	7.69 ± 0.5	6.46 ± 0.50	6.33 ± 0.27	6.47 ± 0.41	6.81 ± 0.66	6.39 ± 0.55	0.523
CREA (μmol/L)	29.00 ± 2.0	37.50 ± 4.35	22.40 ± 3.23^†^	25.00 ± 1.78	29.80 ± 3.92	24.60 ± 3.80	20.25 ± 2.87^†^	**0.033**
HCO_3_^−^ (mmol/L)	21.00 ± 0.63	25.50 ± 3.28	22.60 ± 1.21	22.75 ± 1.55	21.40 ± 1.99	19.40 ± 1.69	22.00 ± 1.47	0.395
Cl^−^ (mmol/L)	96.60 ± 1.63	112.00 ± 9.06	98.20 ± 2.84	103.80 ± 3.00	102.00 ± 1.45	95.60 ± 4.27	96.25 ± 3.61	0.111
K^+^ (mmol/L)	6.59 ± 0.72	7.61 ± 1.31	4.97 ± 0.38	6.17 ± 0.70	7.66 ± 1.00	5.30 ± 0.47	5.54 ± 0.22	0.092
Na^+^ (mmol/L)	136.8 ± 2.94	160.25 ± 12.42	138.6 ± 4.0	145.30 ± 2.50	142.8 ± 2.67	132.00 ± 7.15	133.50 ± 6.59	0.061
Liver function test								
ALT (IU/L)	67.20 ± 7.89	87.75 ± 15.13	63.20 ± 8.32	51.75 ± 4.37	78.60 ± 16.18	12.26 ± 2.27	13.08 ± 6.25	0.401
AST (IU/L)	248.20 ± 31.57	311 ± 34.28	316.8 ± 21.44	268.00 ± 30.00	329.00 ± 77.16	284.60 ± 83.98	263.25 ± 96.41	0.950
ALP (IU/L)	255.80 ± 16.40	290 ± 28.77	278.00 ± 44.76	256.50 ± 33.90	371.60 ± 30.15	214.00 ± 27.72	284.75 ± 56.56	0.097
TBIL (μmol/L)	1.80 ± 0.37	1.75 ± 0.63	1.00 ± 0.63	2.00 ± 0.40	1.40 ± 0.24	1.40 ± 0.40	1.25 ± 0.48	0.760
DBIL (μmol/L)	0.72 ± 0.13	0.35 ± 0.22	0.26 ± 0.09	0.55 ± 0.17	0.30 ± 0.10	0.24 ± 0.10	0.23 ± 0.05	0.068
TP (g/L)	62.00 ± 2.39	75.00 ± 6.10	58.20 ± 2.30	61.50 ± 0.90	61.40 ± 3.49	55.80 ± 3.87^†^	56.00 ± 5.94^†^	**0.037**
ALB (g/L)	29.40 ± 1.29	34.50 ± 3.71	26.40 ± 1.29	27.75 ± 1.03	27.80 ± 1.83	24.45 ± 1.91	25.50 ± 3.43	0.074

*Note:* Data presented as mean ± SEM. ALB = albumin; Cl^−^ = chloride; CREA = creatinine; HCO_3_^−^ = bicarbonate; K^+^ = potassium; Na^+^ = sodium. The bold values indicate statistical significance.

Abbreviations: ALP = alkaline phosphatase; ALT = alanine transaminase; AST = aspartate transaminase; DBIL = direct bilirubin; TBIL = total bilirubin; TP = total protein.

^†^ *p* < 0.05 compared with the control group.

**Table 5:** Mean PSA and testosterone levels among the various groups.

Analyte/unit	Control	Model	*Cm* 30 mg/kg b.wt.	*Cm* 300 mg/kg b.wt.	Tamsulosin/*Cm* 30 mg/kg b.wt.	Tamsulosin/*Cm* 300 mg/kg b.wt.	Tamsulosin	*p* value
PSA (ng/mL)	0.63 ± 0.03	0.55 ± 0.04	0.44 ± 0.03⁣^∗^	0.55 ± 0.06	0.43 ± 0.04⁣^∗^	0.59 ± 0.03	0.50 ± 0.04	**0.006**
Testosterone (nmol/L)	6.9 ± 0.8	154.6 ± 7.5⁣^∗^	169.5 ± 10.5⁣^∗^	143.1 ± 8.5⁣^∗^	170.5 ± 25.7⁣^∗^	167.2 ± 11.8⁣^∗^	161.6 ± 9.6⁣^∗^	**< 0.001**

*Note:* The bold values indicate statistical significance.

⁣^∗^*p* < 0.05 compared with the control group.

^†^ *p* < 0.05 compared with the model group.

## Data Availability

The data that support the findings of this study are available on request from the corresponding author. The data are not publicly available due to privacy or ethical restrictions.

## Nomenclature

BPH: Benign prostatic hyperplasia
LUTS: Lower urinary tract symptoms
PCa: Human prostate cancer
QoL: Quality of life
CM: *Croton membranaceus*
MSCs: Mesenchymal stem cells
PSA: Prostate-specific antigen
tPSA: Total prostate-specific antigen
LD: Low dose
HD: High dose
IPSS: International Prostate Symptom Score
PIS: Prostatic inflammatory status
DHT: Dihydrotestosterone
